# Dataset on the decolorization of Naphthol Green B using a UV/sulfite system: Optimization by response surface methodology

**DOI:** 10.1016/j.dib.2024.110924

**Published:** 2024-09-10

**Authors:** Juan Miguel E. Caguiat, Eldric Roland U. Tiu, Adrian D. Go, Francis M. dela Rosa, Eric R. Punzalan

**Affiliations:** a Department of Chemistry, College of Science, De La Salle University, 2401 Taft Ave, Malate, Manila 1004, Metro Manila, Philippines; b Department of Chemistry, College of Science, Adamson University, 900 San Marcelino St, Ermita, Manila 1000, Metro Manila, Philippines

**Keywords:** Response surface methodology, Advanced oxidation/reduction process, UV/sulfite, Naphthol Green B (NGB)

## Abstract

Naphthol Green B (NGB) is a synthetic azo dye widely used in various industries, including textiles and leathers. NGB poses significant environmental and ecological concerns when released into natural water systems. This paper investigates the decolorization of NGB using UV/sulfite system. The % decolorization of NGB was optimized using 3^2^ Full Factorial Design (FFD), and the ANOVA results show that the model has a good fit for the data (R^2^ = 99.54 %, R^2^_(adj)_ = 98.76 %) and the significant factors contributing to the % decolorization are A, B, A^2^, and B^2^ where *A* = mM sulfite and *B* = pH. The model predicted ≥100 % decolorization with the optimum conditions 12 mM sulfite and pH 10. An actual experiment was conducted to verify the response, resulting in 96.2 % decolorization which is in good agreement with the model.

Specifications Table

**Table:** 

Subject	Environmental Science
Specific subject area	Water Science and Technology
Type of data	Table, Image, Graph, Figure Raw, Analyzed,
Data collection	The % decolorization of NGB was evaluated determining its absorbance after UV illumination. 3^2^ Full Factorial Design (FFD) was used to the pH (4–10) and sulfite concentration (4–12 mM). Verification of the model was simulated using the predicted optimum conditions (12 mM sulfite and pH 4). An aliquot of 3 mL was obtained every 10 mins and the extent of decolorization were evaluated via UV–vis spectroscopy (λ_max_ = 714 nm). MINITAB 17 software was used to generate the response model.
Data source location	*· Institution: De La Salle University* *· City/Town/Region: Manila* *· Country: Philippines* *· Coordinates for collected data: 14.565377948974492, 120.9923349203574*
Data accessibility	Repository name: Mendeley Data Data identification number: Go, Adrian (2024), “Dataset on the Decolorization of NGB using a UVSulfite system Optimization by RSM V2”, Mendeley Data, V2, doi: 10.17632/p6dk46nmpx.2 Direct URL to data: https://data.mendeley.com/datasets/p6dk46nmpx/2

## 1 Value of the Data

• The results of the full factorial design (FFD) provide a robust descriptor of the optimum pH and sulfite concentration, which can lead to more refined studies involving a broader range of values.
• Researchers can test and compare the effects of experimental factors used in this dataset (sulfite concentration and pH) on the effectiveness of the sulfite system in the decolorization of other related organic compounds.
• The data demonstrates the potential of sodium sulfite as a viable solution for industrial wastewater treatment, particularly for the decolorization of complex dyes.
• This dataset adds critical information for the decolorization of transition-metal centered dyes, enhancing the existing body of knowledge. It facilitates further research in wastewater treatment and the development of more effective methods for treating industrial effluents containing such dyes.
• This comprehensive dataset serves as a valuable resource for developing machine learning algorithms and AI prediction models. These models can help predict decolorization efficiency of different dyes and optimize experimental conditions for similar applications.

## 2 Background

The utilization of UV/sulfite for the decolorization of Naphthol Green B dye presents a sustainable approach for wastewater treatment in industrial settings. This dataset was compiled to help researchers systematically analyze the influence of various parameters on sulfite-mediated decolorization using response surface methodology, thereby enhancing decolorization efficiency.

Additionally, the generated data provides valuable insights for data mining specialists. Researchers can identify patterns and correlations from the experimental results to gain a deeper understanding of the decolorization mechanism and its influencing factors. This data can also be leveraged for predictive modelling and enable the estimation of decolorization efficiency under different conditions.

## 3 Data Description

**Fig. 1:**
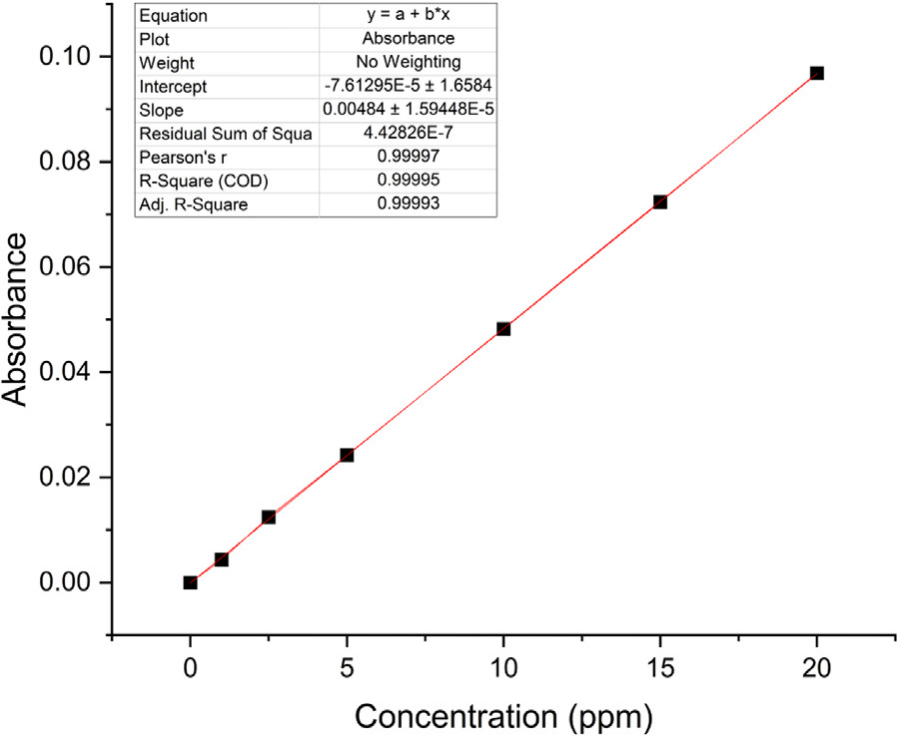
NGB concentration standard curve.

**Fig. 2:**
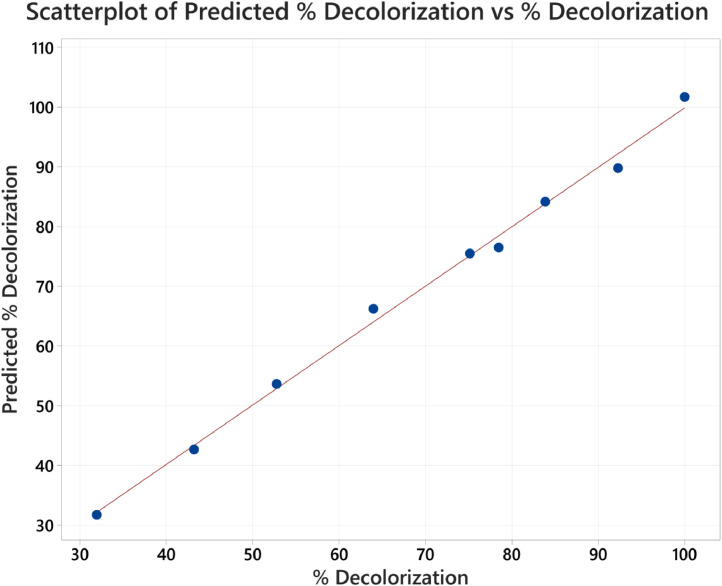
Predicted vs Actual % Decolorization of NGB.

**Fig. 3:**
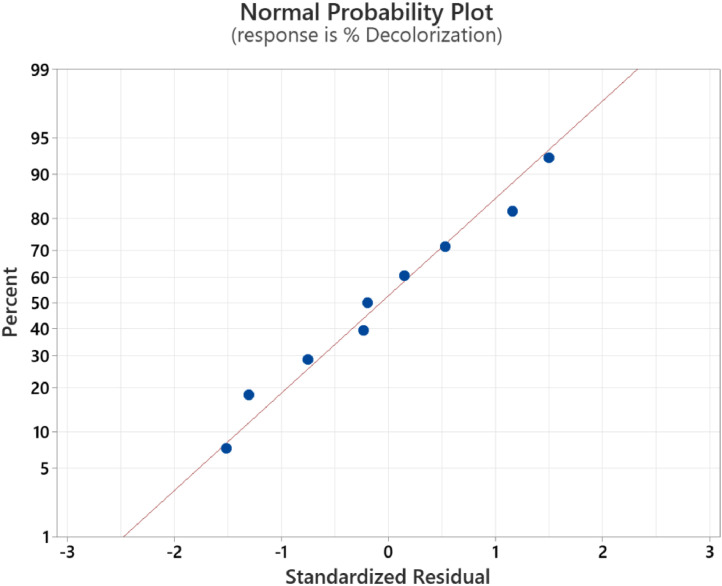
Normal probability plot of standardized residuals.

**Fig. 4:**
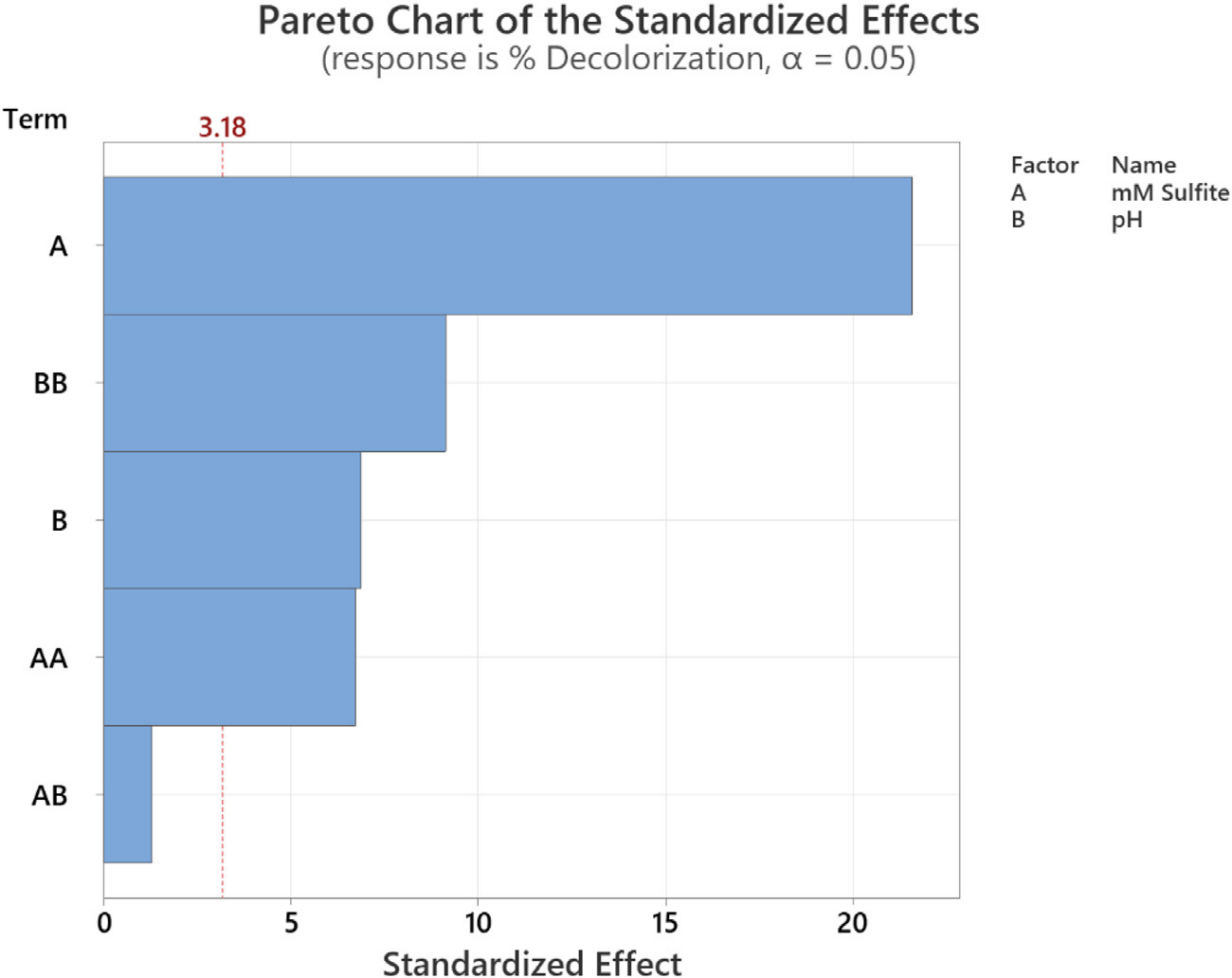
Pareto chart of standardized effects.

**Fig. 5:**
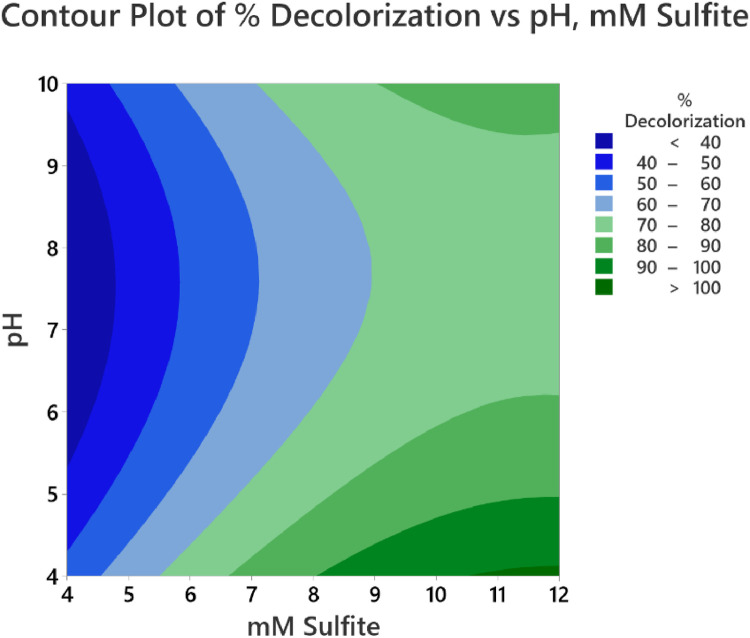
Contour plot of NGB decolorization.

**Fig. 6:**
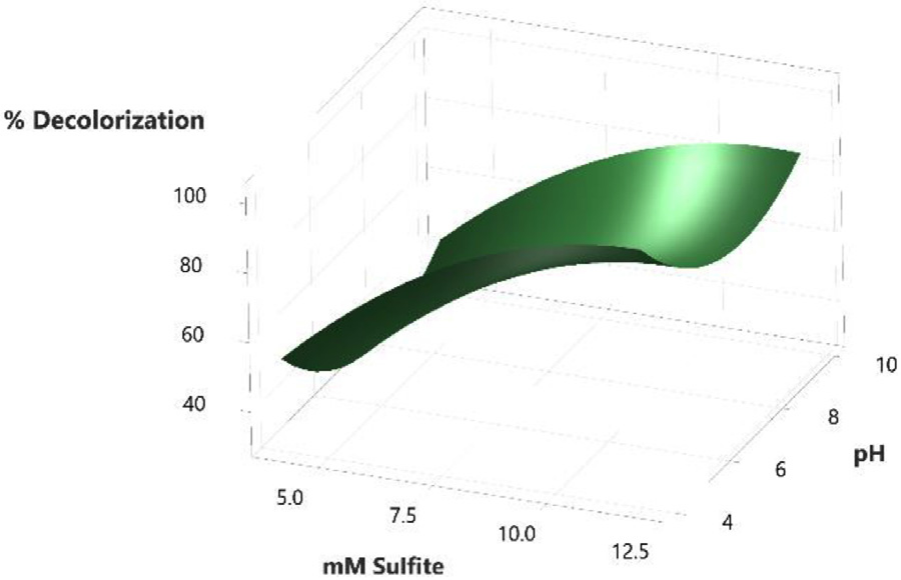
Surface plot of NGB decolorization.

**Fig. 7:**
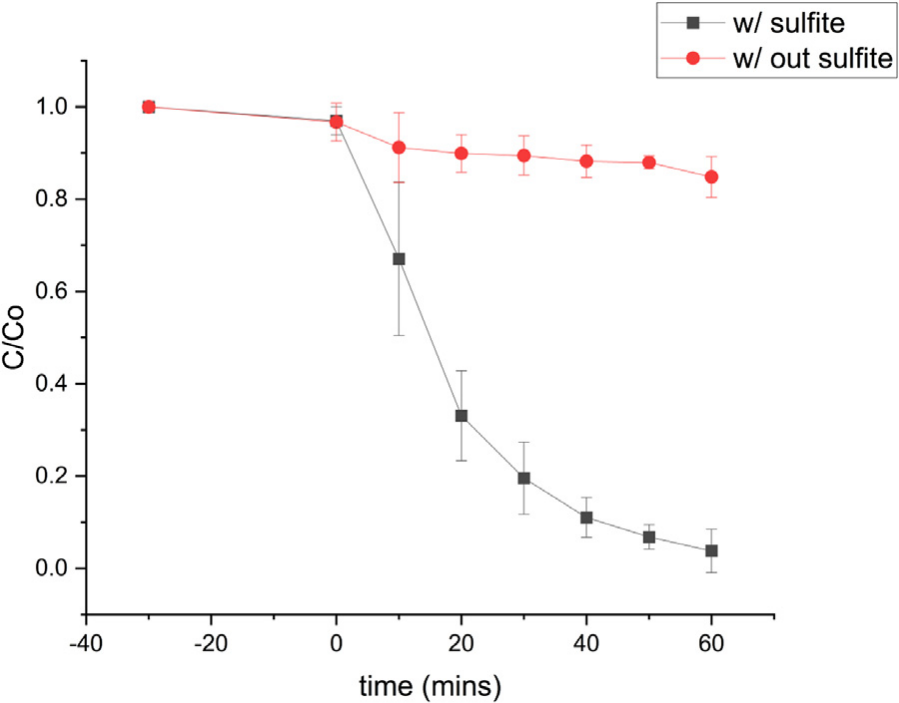
Verification of NGB decolorization at 12 mM sulfite and pH 4 over 60 min.

The calibration curve for standard solutions of NGB concentration (1–20 ppm) was created. The measured absorbance was plotted against the concentration shown in Fig. 1. Pearson's coefficient (R) for the calibration curve was determined to be 0.999 which signifies a strong linear relationship at this concentration range. The decolorization of NGB using UV/sulfite was optimized using a 3^2^ Full Factorial Design (FFD) to determine the optimum combination of sulfite and pH concentration. The range and levels of FFD design are presented in Table 1 and the parameters assigned to the model were (A) mM sulfite and (B) pH. The design table (Table 2) used 9 base runs, each being a unique combination of sulfite concentration values of 4, 8, and 12 mM and pH values of 4, 7, and 10. Whereas, the % decolorization of NGB after 60 min of UV illumination was recorded as the response. The graphical plot of predicted % decolorization against actual % decolorization response is presented in Fig. 2. The variables that have a significant effect on the % decolorization are identified using the analysis of variance (ANOVA). In addition, ANOVA is also used to assess the modelʼs predictability and level of fit. The significant variables for the model are identified using the Fisher (F) and probability (P) values. Model terms are significant when "Prob > *F*" values are less than 0.0500; otherwise, they are not significant [1]. The evaluated equation form shown in Table 3 suggest that quadratic equation best describes the model (highest R values). The generated regression quadratic equation is shown in Eq. (1). Based on the ANOVA data in Table 4, the significant variables contributing to the model response are A, B, A^2^, and B^2^. However, the interaction between the mM sulfite and pH-(AB) are not significant [2]. The coefficients of determination (R^2^) and adjusted R^2^ have values near 1 which is indicative of a good model fitting and appropriateness. The normal probability plot of standardized residuals (Fig. 3) follows a linear trend which indicates normally distributed data points. The magnitude and significant factors are shown in pareto chart (Fig. 4). Based on the pareto chat, the most influential effect is **A**, followed by **B^2^, B**, and **A**. Whereas, the factor **AB** is not statistically significant at 0.05 level. The contour and surface interaction plots of the variable are shown in Fig. 5, Fig. 6, respectively. The graph shows that % decolorization favors higher mM sulfite concentration at acidic conditions (low pH). Using the Minitab software, response optimization was set to the maximum response which generated the predicted % decolorization of ≥100 % at conditions; 12 mM sulfite and pH 4. The parameters were simulated in actual experiments shown in Fig. 7. The actual % decolorization was determined to be 96.2 %, which closely matches the predicted response.

(1) $$\%\;\text{Decolorization}=66.20+22.40A-7.13B-12.09A^{2}+16.41B^{2}-1.65\text{AB}$$

**Table 1:** Range and levels of full factorial design.

Variable	Low (−1)	Center (0)	High (+1)
(A) mM sulfite	4	8	12
(B) pH	4	7	10

**Table 2:** 3^2^ Full factorial design table.

Std. Order	mM sulfite	pH	% Decolorization	Predicted % Decolorization
1	4	4	52.8	53.6
2	12	4	100.0	101.7
3	4	10	43.2	42.6
4	12	10	83.9	84.1
5	4	7	32.0	31.7
6	12	7	78.5	76.5
7	8	4	92.3	89.7
8	8	10	75.2	75.5
9	8	7	64.0	66.2

**Table 3:** Evaluated equation form.

Model	R^2^	Adjusted R^2^	Predicted R^2^
Linear	79.38 %	72.50 %	60.33 %
2-Factor Interaction	79.63 %	67.42 %	60.11 %
Quadratic	99.54 %	98.76 %	95.59 %

**Table 4:** ANOVA table.

Source	DF	Adj SS	Adj MS	F-Value	*P*-Value	Remarks
Model	5	4156.64	831.33	128.52	0.001	significant
Linear	2	3314.76	1657.38	256.22	0.000	significant
mM Sulfite	1	3009.63	3009.63	465.27	0.000	significant
pH	1	305.13	305.13	47.17	0.006	significant
Square	2	831.05	415.53	64.24	0.003	significant
mM Sulfite*mM Sulfite	1	292.36	292.36	45.20	0.007	significant
pH*pH	1	538.69	538.69	83.28	0.003	significant
2-Way Interaction	1	10.83	10.83	1.67	0.286	not significant
mM Sulfite*pH	1	10.83	10.83	1.67	0.286	not significant
Error	3	19.41	6.47			
S	2.54334					
R^2^	99.54 %					
R^2^ (adj)	98.76 %					
R^2^ (pred)	95.59 %

## 4 Experimental Design, Materials and Methods

### 4.1 Materials

The materials used for the study were sodium sulfite (Na_2_SO_3_, ≥98.0 %, Sigma Aldrich), Naphthol Green B (Na_3_[Fe(C_10_H_5_NO_5_S)_3_]) (Xilong Scientific), sodium hydroxide (NaOH, 97 %, Sigma Aldrich) and hydrochloric Acid (HCl, 97 %, Sigma Aldrich). The instrument used for the UV–vis spectroscopy analysis was Hitachi U-2900 spectrophotometer.

### 4.2 Calibration curve

A calibration curve for NGB was constructed to accurately determine the concentration of NGB from the UV–vis spectroscopy signal. Standard concentrations of 1, 2.5, 5, 10, 15, and 20 ppm NGB (λ_max_ = 714 nm) were prepared. The absorbance of the prepared standards was measured using UV–vis spectrophotometer.

### 4.3 Determination of NGB % decolorization

The photoreactor box used was custom made with a 254 nm UV light placed approximately 10 cm above the working solution. A water pump system was added and connected to jacketed beakers to maintain constant temperature [3,4]. Stock solution for the experiment was prepared by dissolving NGB in distilled water in a 1 L volumetric flask and diluting to mark. Additional dilutions were performed from the stock solution to obtain the desired concentration of NGB. A mass of sodium sulfite was weighed and transferred to mix with 100 mL of NGB solution. Afterwards, the working solution was transferred into a double-layered beaker. The pH of the solutions was adjusted with a dropwise addition of diluted HCl or NaOH until the pH was of the desired value. The solution was allowed to stir for 30 min in the dark before opening the UV light. Upon UV-illumination, an aliquot was obtained every 10 min and had its absorbance measured by UV–vis spectrophotometer at 714 nm [5]. The % decolorization was calculated using the following equation,

(2) $$\%\;\text{Decolorization}=\frac{C_{o}-C}{C_{o}}x100\%$$

Where C_o_ = initial concentration and C = variable concentration

### 4.4 Optimization

Minitab® was the software used for the model analysis. A 3^2^ Full Factorial Design (FFD) was used for the optimization of the decolorization of NGB. The design table is composed of 9 runs with parameters: (A) sulfite dose (mM), and (B) pH. The response for the model was % decolorization of NGB after 60-minute illumination. The predicted optimum parameters for the % decolorization were generated by setting the response to maximum.

### 4.5 Verification of response model

The predicted parameters from the model were determined to be 12 mM sulfite and pH 4. An experiment was conducted according to the predicted parameters. An aliquot (3 mL) of the NGB solution was obtained per 10-minute interval during the UV illumination. Afterwards, NGB concentration was evaluated using a UV–vis spectrophotometer.

## Limitations

Not applicable.

## Ethics Statement

The authors confirm that they have read and adhere to the ethical requirements for publication. The current work does not involve human subjects, animal experiments, or any data collected from social media platforms.

## CRediT Author Statement

**Juan Miguel Caguiat**: Investigation, Methodology, Writing, Editing. **Eldric Roland Tiu**: Investigation, Methodology. **Adrian Go**: Resources, Review, Editing. **Francis dela Rosa**: Visualization, Editing, Review. **Eric Punzalan**: Supervision, Review.

## Data Availability

Dataset on the Decolorization of NGB using a UVSulfite system Optimization by RSM V2 (Original data) (Mendeley Data).
